# Altered phobic reactions in frontotemporal dementia: A behavioural and neuroanatomical analysis

**DOI:** 10.1016/j.cortex.2020.05.016

**Published:** 2020-09

**Authors:** Daniel A. Jimenez, Rebecca L. Bond, Mai-Carmen Requena-Komuro, Harri Sivasathiaseelan, Charles R. Marshall, Lucy L. Russell, Caroline Greaves, Katrina M. Moore, Ione OC. Woollacott, Rachelle Shafei, Chris JD. Hardy, Jonathan D. Rohrer, Jason D. Warren

**Affiliations:** a Dementia Research Centre, UCL Queen Square Institute of Neurology, University College London, London, United Kingdom; b Department of Neurological Sciences, Faculty of Medicine, University of Chile, Santiago, Chile; c Preventive Neurology Unit, Wolfson Institute of Preventive Medicine, Queen Mary University of London, London, United Kingdom

**Keywords:** Frontotemporal dementia, Alzheimer's disease, Specific phobia, Emotion, Fear, Neuroimaging, VBM

## Abstract

**Introduction:**

Abnormal behavioural and physiological reactivity to emotional stimuli is a hallmark of frontotemporal dementia (FTD), particularly the behavioural variant (bvFTD). As part of this repertoire, altered phobic responses have been reported in some patients with FTD but are poorly characterised.

**Methods:**

We collected data (based on caregiver reports) concerning the prevalence and nature of any behavioural changes related to specific phobias in a cohort of patients representing canonical syndromes of FTD and Alzheimer's disease (AD), relative to healthy older controls. Neuroanatomical correlates of altered phobic reactivity were assessed using voxel-based morphometry.

**Results:**

46 patients with bvFTD, 20 with semantic variant primary progressive aphasia, 25 with non-fluent variant primary progressive aphasia, 29 with AD and 55 healthy age-matched individuals participated. Changes in specific phobia were significantly more prevalent in the combined FTD cohort (15.4% of cases) and in the bvFTD group (17.4%) compared both to healthy controls (3.6%) and patients with AD (3.5%). Attenuation of phobic reactivity was reported for individuals in all participant groups, however new phobias developed only in the FTD cohort. Altered phobic reactivity was significantly associated with relative preservation of grey matter in left posterior middle temporal gyrus, right temporo-occipital junction and right anterior cingulate gyrus, brain regions previously implicated in contextual decoding, salience processing and reward valuation.

**Conclusion:**

Altered phobic reactivity is a relatively common issue in patients with FTD, particularly bvFTD. This novel paradigm of strong fear experience has broad implications: clinically, for diagnosis and patient well-being; and neurobiologically, for our understanding of the pathophysiology of aversive sensory signal processing in FTD and the neural mechanisms of fear more generally.

## 1 Introduction

Fear is an emotion of fundamental biological importance. Evolutionarily ancient, it signals danger, directs actions that preserve life and limb and thereby promotes survival. As a subjectively unpleasant state, avoidance of fear motivates learning and adaptive behaviour that ultimately enhances wellbeing. Though related to anxiety, fear is distinct phenomenologically, clinically and biologically (Robinson, Pike, Cornwell, & Grillon, 2019): whereas anxiety is a diffuse and pervasive response to chronic, potential or uncertain threat that is typically accompanied by passive avoidance, fear is a phasic response to specific and imminent danger and mobilises immediate and active avoidance behaviour. In further contrast to anxiety, fear in undiluted form is rarely experienced by adult humans under ordinary conditions in everyday life - while to engender it deliberately is generally ethically unacceptable. This is a fortunate state of affairs but also makes the experience of fear difficult to study experimentally. It presents a particularly pertinent challenge in neurodegenerative diseases, notably the frontotemporal dementias (FTD), in which altered emotion processing is a leading clinical issue and potentially a core pathophysiological principle (Kumfor & Piguet, 2012; Lyketsos et al., 2000; Marshall et al., 2019; Rascovsky et al., 2011; Sivasathiaseelan et al., 2019).

Multimodal impairments of emotion decoding and homeostatic signal processing underpinned by fronto-temporo-limbic circuit dysfunction are increasingly recognised in FTD and may contribute to loss of empathy and aberrant socio-emotional reactivity (Farb et al., 2013; Kumfor & Piguet, 2012; Marshall, Hardy, Allen, et al., 2018; Marshall et al., 2017, 2019; Marshall, Hardy, Russell, et al., 2018; Omar et al., 2011). In Alzheimer's disease (AD), emotion processing deficits tend to be less prominent but are increasingly also recognised even at earlier disease stages and adversely affect clinical outcomes (Barnes et al., 2015; Lyketsos et al., 2011). The role of altered emotionality in reward seeking, affective learning and other complex behaviours exhibited by patients with FTD and AD has received much recent attention (Clark et al., 2018; Cohen et al., 2016; Fletcher et al., 2015a, Fletcher et al., 2015b; Hua et al., 2018; Sturm et al., 2017). However, while there is some evidence for abnormal emotional learning based on fear conditioning in FTD and AD (Hoefer et al., 2008), ‘primitive’ emotions such as fear remain poorly understood and comparatively little studied in these diseases. Alterations of fear processing are not probed by the Neuropsychiatric Inventory (NPI), the most widely validated instrument for evaluating neuropsychiatric and behavioural symptoms in people with dementia (Cummings et al., 1994). Previous work on fear processing in dementia has largely focussed on recognition of fear as a ‘universal’ emotion, conveyed by the facial, vocal or bodily expressions of other people (Bora, Velakoulis, & Walterfang, 2016; Keane, Calder, Hodges, & Young, 2002; Kumfor et al., 2014; Kumfor, Irish, Hodges, & Piguet, 2013; Kumfor & Piguet, 2012; Omar et al., 2011; Rohrer, Sauter, Scott, Rossor, & Warren, 2012; Torres Mendonça De Melo Fádel, Santos De Carvalho, Belfort Almeida Dos Santos, & Dourado, 2019; Van den Stock et al., 2015). While this work has demonstrated impaired recognition of fear and other negative emotions in FTD syndromes and (less prominently and consistently) in AD, it does not address the subjective experience of fear in dementia, which is likely to be more relevant in responding to acute threats and behaving adaptively in the world at large.

A candidate model system for studying the experience of strong fear and related behaviours under ‘natural’ conditions may be to hand, in the phenomenon of phobias. Phobias comprise a group of disorders recognised by the Diagnostic and Statistical Manual of Mental Disorders (DSM)-V (American Psychiatric Association, 2013). Specific phobia, the most common form, can be considered a focal presentation of abnormal fear reactivity: it is characterised by disproportionate fear and anxiety in response to a very well circumscribed phobic object or situation, most commonly animals or heights (Stinson et al., 2007), leading to active avoidance by the phobic individual. Though variable in severity, specific phobia is relatively common in the general population, with an overall lifetime prevalence estimated at around 9%; female sex, younger age and low socio-economic status are associated with a relatively higher risk of exhibiting specific phobia (Sigström et al., 2011; Stinson et al., 2007). Phobias constitute one of the few instances of frequent, reproducible and powerful fear experiences in everyday life, and might therefore open a window on fear processing mechanisms and behaviours in clinical settings, including dementia.

A modulatory role of ageing on phobic reactivity is suggested by epidemiological evidence for an age-related decline in the prevalence and severity of specific phobia (Byers, Yaffe, Covinsky, Friedman, & Bruce, 2010; Chou et al., 2011; Grenier et al., 2019; Sigström, Skoog, Karlsson, Nilsson, & Östling, 2016; Stinson et al., 2007). Attenuated phobic responses have been reported in the semantic variant of primary progressive aphasia (svPPA) (Clark et al., 2014) while anecdotally, clinical experience suggests that alterations of phobic reactivity (variably heightened or attenuated) occur not uncommonly in the wider FTD spectrum. However, this clinical impression remains to be substantiated; even in the healthy population, phobias have not been well studied, especially among older people (Grenier et al., 2011; Stinson et al., 2007). One important biological rationale for assessing phobic reactivity in dementia syndromes is the neuroanatomy of phobic responses: available evidence in the healthy brain has implicated a distributed network of brain regions in the generation of specific phobic responses, including amygdala, insula, medial prefrontal and extrastriate visual cortices (Caseras et al., 2010; Del Casale et al., 2012; Ipser, Singh, & Stein, 2013; Linares et al., 2014, 2012; Mobbs et al., 2010; Stefanescu, Endres, Hilbert, Wittchen, & Lueken, 2018; Wabnegger, Scharmüller, & Schienle, 2014). These areas closely overlap the core brain networks targeted in canonical syndromes of FTD and AD (Mahoney et al., 2015; Marshall et al., 2019; Raj, Kuceyeski, & Weiner, 2012; Seeley, Crawford, Zhou, Miller, & Greicius, 2009; Sivasathiaseelan et al., 2019; Warren et al., 2013; Warren, Fletcher, & Golden, 2012; Zhou, Gennatas, Kramer, Miller, & Seeley, 2012), suggesting that studying phobic responses in dementias may illuminate our understanding of the neural mechanisms critical for mediating phobic reactivity in health as well as neurodegenerative disease.

Here, we used specific phobia as a model paradigm for assessing the prevalence and phenomenology of altered phobic (or more generally, subjective fear) reactivity in a well characterised cohort of patients representing major phenotypes of FTD (behavioural variant FTD (bvFTD)), svPPA and the nonfluent-agrammatic variant of primary progressive aphasia (nfvPPA), in relation to patients with AD and healthy older individuals. Structural neuroanatomical associations of phobic changes were assessed using voxel based morphometry (VBM). We hypothesised that altered phobic reactivity would be more prevalent in FTD than in AD and the healthy older population and would manifest as a complex spectrum of heightened and attenuated phobic responses. We further hypothesised that altered phobic reactivity would correlate with grey matter changes in brain regions previously implicated in the generation of phobic responses, in particular amygdala, insula, anterior cingulate and occipito-temporal junctional cortices (Caseras et al., 2010; Del Casale et al., 2012; Ipser et al., 2013; Linares et al., 2014, 2012; Mobbs et al., 2010; Stefanescu et al., 2018; Wabnegger et al., 2014).

## 2 Material and methods

In this section, we report how we determined our sample sizes, all data exclusions, all inclusion and exclusion criteria, whether these criteria were established prior to data analysis, all manipulations and all measures in the study.

### 2.1 Participant characteristics

Consecutive patients with the target dementia diagnoses were recruited via the Specialist Cognitive Disorders Clinics at the National Hospital for Neurology and Neurosurgery and healthy older individuals via the Dementia Research Centre research volunteer database. Ninety-one patients with syndromes of FTD (34 female, aged 66.1 ± 7.1 years) comprising 46 patients with bvFTD, 25 with nfvPPA and 20 with svPPA, 29 patients with a typical amnestic presentation of Alzheimer's disease (15 female, aged 70.9 ± 7.8 years) and 55 healthy individuals (25 female, aged 64.9 ± 7.3 years) participated. These sample sizes were determined to be sufficient to detect likely overall group effect sizes, based on empirical observations in other phenomenological studies involving this cohort and supported by formal power calculations. All patients fulfilled inclusion criteria for the study established prior to data analysis, i.e., consensus diagnostic criteria for the relevant syndrome (Gorno-Tempini et al., 2011; McKhann et al., 2011; Rascovsky et al., 2011), of mild to moderate severity and further corroborated by clinical neuropsychometry, brain MRI, CSF biomarkers and/or genetic testing. Genetic screening revealed pathogenic mutations in 19 patients with bvFTD (eight *C9orf72*, seven *MAPT*, four *GRN*), one patient with svPPA (*MAPT*), four patients with nfvPPA (*GRN*) and one patient with AD (*PSEN2*). Exclusion criteria for the study, established prior to data analysis, were a significant comorbid cerebrovascular disease burden on MRI; or (based on a detailed history corroborated by patients' primary caregivers) a past (premorbid) history of a generalised anxiety disorder, major affective, psychotic or other intercurrent psychiatric disorder (excepting specific phobia). No potential participants required exclusion based on these criteria. Demographic and clinical details of participant groups are summarised in Table 1.

**Table 1:** Summary of demographic, clinical and phobic reactivity data for participant groups.

Characteristic	Controls	bvFTD	svPPA	nfvPPA	AD
**General clinical**					
No. (female: male)	25:30	13:33	9:11	12:13	15:14
Age, years	64.9 (7.3)	64.5 (6.3)	65.8 (7.1)	69.3 (7.7)	70.9 (7.8)^b,c^
Symptom duration, years	NA	6.8 (4.7)	5.5 (2.3)	4.8 (4.5)	6.7 (3.4)
MMSE (/30)	29.4 (.9)^a^	24.2 (5.6)^b^	23.7 (6.0)^b^	20.8 (7.9)^b,c^	19.2 (6.0)^b,c^
**General neuropsychiatric symptoms**^d^					
Apathy, n (%)	2 (4.9)^a^	37 (88.1)^b^	9 (47.4)^b,c^	14 (60.9)^b,c^	20 (69.0)^b^
Hallucinations, n (%)	0	11 (26.2)	1 (5.3)	0	4 (13.8)
Delusions, n (%)	0	17 (40.5)	4 (21.1)	3 (13.0)^c^	4 (13.8)^c^
Anxiety, n (%)	3 (7.3)^a^	19 (45.2)^b^	11 (57.9)^b^	17 (73.9)^b,c^	16 (55.2)^b^
Agitation, n (%)	0	16 (38.1)	4 (21.1)	3 (13.0)^c^	2 (6.9)^c^
Altered self-boundaries, n (%)	0	9 (37.5)	5 (26.3)	3 (15)	3 (10.3)^c^
**Phobic reactivity**					
Altered phobic reactions (any), n (%):	2 (3.6)	8 (17.4)	3 (15)	3 (12)	1 (3.5)
OR (95% CI) versus healthy controls^e^	NA	5.6 (1.1–28.2)^b^	4.6 (.7–30.0)	3.1 (.5–20.4)	.8 (.1–9.2)
*Acquired new phobia, n (%)*	0	4 (8.7)	2 (10)	2 (8)	0
*Loss of previous phobia, n (%)*	2 (3.6)	4 (8.7)	1 (5)	1 (4)	1 (3.5)

Mean (SD) values are shown unless otherwise indicated. Key: ^a^significantly different from disease groups, *p* < .05; ^b^significantly different from healthy control group, *p* < .05; ^c^significantly different from bvFTD group, *p* < .05; ^d^of any severity (see text and Table S1); ^e^logistic regression adjusted for age and gender; AD, patient group with typical Alzheimer's disease; bvFTD, patient group with behavioural variant frontotemporal dementia; CI, 95% confidence interval; Controls, healthy control group; MMSE, Mini-Mental State Examination score; NA, not applicable; nfvPPA, patient group with non-fluent variant primary progressive aphasia; OR, odds ratio; SD, standard deviation; svPPA, patient group with semantic variant primary progressive aphasia.

The study was approved by the institutional ethics review board, and all participants gave written informed consent in line with Declaration of Helsinki guidelines. No part of the study procedures or analyses was pre-registered prior to the study being conducted. The conditions of our ethics approval do not permit public archiving of anonymised study data. Readers seeking access to the data should contact the corresponding author; access will be granted to named individuals in accordance with ethical procedures governing the reuse of clinical data, including completion of a formal data sharing agreement and approval of the local ethics committee.

### 2.2 General neuropsychiatric assessment

In order to provide a background neuropsychiatric context for phobic alterations in the participant groups, we collected data on the prevalence (presence/absence) of general neuropsychiatric symptoms for patients and healthy controls using a survey questionnaire (presented in Table S1 in Supplementary Material online). The survey was completed by the patient's primary caregiver or by healthy controls themselves; we asked whether any of the surveyed symptoms was currently present, with illustrative examples of each symptom. We assessed the prevalence of those behavioural symptoms anticipated to be potentially relevant to the development and/or expression of altered phobic reactivity, namely apathy, hallucinations or delusions, anxiety, agitation and altered boundaries of self (e.g., dislike of being approached or touched by others). If a history of psychotic symptoms (delusions or hallucinations) was volunteered, we established whether these in any way involved the phobic object, with the intention to exclude any such cases from the further phobic reactivity analysis; no participant required exclusion on this basis.

### 2.3 Documentation of altered phobic reactivity

From the primary caregiver of each participating patient and from each of the healthy controls, we recorded (see Table S1), if the participant had ever reported or exhibited evidence of a specific phobia (defined in line with current DSM-5 criteria for ‘specific phobia’ (American Psychiatric Association, 2013) as an intense, unreasonable fear consistently in response to a specific object or situation, disproportionate to actual danger and which had led the person to try to avoid the object or situation): i) the nature of the phobic object or situation; ii) whether there had been any change in the nature or intensity of the phobic reaction within the past 10 years; and iii) if there had been a change, the direction of this (increased or diminished reactivity). All phobic stimuli were recorded if more than one triggering object or situation was reported. Participants and caregivers were also invited to make any additional comments about the phobia, which we also recorded. Cases with phobic symptoms that could reflect social anxiety (formerly ‘social phobia’) or a generalised anxiety disorder were excluded, as these are likely to overlap with general behavioural symptoms in the target diseases and may not reflect altered phobic reactivity per se.

### 2.4 Analysis of demographic, clinical and behavioural data

Participants' demographic and clinical data and phobic reaction report data were analysed using STATA version 14.0 software (StataCorp, College Station, TX, USA). Summary statistics are presented for selected variables in healthy controls and patients grouped as AD, FTD and FTD syndromic subgroups. Overall differences across groups and between healthy controls and patients were tested using Chi-square and Fisher's exact tests in the case of categorical variables and one-way ANOVA or Kruskal–Wallis H test for continuous variables depending on normality of distribution. We performed post hoc analyses to assess differences between groups where significant overall differences were found. A logistic regression model was fitted to assess the effect of diagnosis as the dependent variable on the risk of altered phobic reactivity, covarying to adjust for potentially confounding variables of age and gender. We calculated summary statistics for demographic and clinical variables in those with and without altered phobic reactivity within each diagnostic group. Finally, we assessed for any association of change in phobic reactivity with general demographic and clinical features (age, gender and in the patient groups, Mini-Mental State Examination (MMSE) score and symptom duration) and with the presence of other neuropsychiatric symptoms (apathy, hallucinations, delusions, anxiety, agitation and altered boundaries of self) using the Spearman rank-order correlation coefficient.

### 2.5 Brain image acquisition and analysis

For the purpose of determining neuroanatomical correlates of altered phobic reactivity, we performed a VBM analysis on the largest syndromic group in our sample. Patients with bvFTD and changes in phobic reactivity were compared with a ‘disease control’ group of bvFTD patients without any reported alteration in phobic reactivity selected from the same bvFTD cohort and matched by age and gender to the symptomatic group.

Brain MR images were acquired for patients in the bvFTD group on a Siemens Prisma or Trio 3T MRI scanner using a 32-channel phased array head-coil and 3-D magnetization-prepared rapid-gradient echo T1-weighted volumetric brain MR sequence (TE/TR/TI 2.9/2200/900 msec, dimensions 256 × 256 × 208, voxel volume of 1.1 × 1.1 × 1.1 mm). MRI images were converted to Neuroimaging Informatics Technology Initiative format and visually reviewed in axial, sagittal and coronal planes for image quality; one scan was excluded due to significant movement artefacts and poor grey: white contrast. The final set of MR images included in the VBM analysis comprised seven cases with bvFTD showing altered phobic reactivity and 19 cases without reported phobic alterations.

These brain images were pre-processed using SPM12 (Statistical Parametric Mapping, Wellcome Trust Centre for Neuroimaging, London, UK) running on Matlab7 (The Mathworks, MA, USA). The scans were rigidly reoriented to standard space and segmented into cerebrospinal fluid, grey and white matter. Grey matter segments were imported for registration to a group-specific space using the DARTEL toolbox, modulated and finally smoothed using a 6 mm full-width-at-half-maximum Gaussian kernel. An automatic thresholded mask was created for grey matter using the smoothed, modulated and warped segments (Ridgway et al., 2009). Total intracranial volume (TIV) was automatically estimated in SPM12 as an index of pre-morbid total grey matter volume (Malone et al., 2015). The whole-brain native-space bias-corrected images obtained from the segmentation were used to generate a mean template brain image on which results were displayed.

Regional grey matter volume differences were modelled as a function of the presence or absence of altered phobic reactivity voxel-wise over the whole brain volume and incorporating age, gender, MRI scanner (Siemens Prisma or Trio) and TIV as covariates of no interest. Grey matter associations of altered phobic reactivity were assessed bidirectionally (i.e., we sought to identify voxels signifying either grey matter atrophy or relative preservation linked to phobic alterations) at an initial ‘cluster-defining’ uncorrected significance threshold *p* < .001; significant local maxima are reported at threshold *p* < .05, after family-wise error (FWE) correction for multiple voxel-wise comparisons within pre-specified anatomical regions of interest. These regions (shown in Figure S1 in Supplementary Material online) were based on our prior neuroanatomical hypotheses (Caseras et al., 2010; Del Casale et al., 2012; Ipser et al., 2013; Linares et al., 2012; Mobbs et al., 2010; Stefanescu et al., 2018) and customised from the Oxford/Harvard brain maps to fit the group mean template brain image: they comprised bilateral insula, amygdala, anterior cingulate cortex and a composite region covering the temporo-occipital junction (including posterior middle temporal gyrus and inferior lateral occipital cortex).

## 3 Results

### 3.1 General characteristics of participant groups

Table 1 summarises general demographic and clinical characteristics of each participant group. Males were over-represented in the healthy control and FTD groups (with the largest gender discrepancy in the bvFTD group), but gender distribution did not differ significantly between groups [*X*^2^ (4,N = 175) = 5.39, *p* = .3]. Age at clinical assessment showed an overall difference across groups [*F* (4,170) = 5.33, *p* < .001], driven by the slightly older age range of the nfvPPA and AD groups. Age and gender were included as covariates of no interest in all subsequent group comparisons. As expected, patient groups had lower mean MMSE scores than the healthy control group [*H* (4) = 73.1, *p* < .001], AD patients showing the worst performance across dementia groups. Syndromic groups also differed significantly in the proportion of cases presenting neuropsychiatric symptoms [*X*^2^ (4,N = 154) = 93.93, *p* < .001]; the bvFTD cohort showed the highest prevalence of neuropsychiatric symptoms. Only anxiety and apathy were reported for healthy controls, albeit with significantly lower frequency than in any disease group [*X*^2^ (1,N = 154) = 90.89, *p* < .001].

### 3.2 Altered phobic reactivity

Characteristics of phobic alterations for each participant group are summarised in Table 1; characteristics of the cohort stratified for presence or absence of altered phobic reactivity are summarised in Table S2 in Supplementary Material online. We identified 17 individuals with a change in phobic reactivity developing within the past 10 years: these comprised 14 patients with FTD syndromes (15.4% of the combined FTD group), one patient with AD (3.5% prevalence) and two healthy controls (3.6% prevalence). The probability of any change in phobic response in the FTD cohort was more than four times higher than in healthy controls, after adjusting for age and gender (odds ratio [OR] 4.6, 95% confidence interval [CI] 1.0 to 21.2, *p* = .050). In contrast, there was no significant difference in the prevalence of phobic changes between AD patients and healthy controls (OR .8, 95% CI .1 to 9.6, *p* = .9). Within the FTD cohort, changes in phobic reactivity were most prevalent in the bvFTD syndromic group (17.4%) and the risk of phobic changes was over five times higher in this group than in healthy controls, after adjusting for age and gender (OR: 5.6, 95% CI 1.1 to 28.2, *p* = .037). The prevalence of phobic alterations in the svPPA and nfvPPA syndromic groups did not differ significantly from healthy controls, however this may simply reflect the smaller sample sizes, as the prevalence rate in each of these other FTD syndromic groups was similar to bvFTD (see Table 1). Altered phobic reactivity was not significantly associated with age (*r*_*s*_ = .05, *p* = .5), gender (*r*_*s*_ = −.05, *p* = .5), symptom duration (*r*_*s*_ = −.02, *p* = .8), MMSE score (*r*_*s*_ = .01, *p* = .9) or other neuropsychiatric symptoms (general anxiety (*r*_*s*_ = .05, *p* = .6)), agitation (*r*_*s*_ = .04, *p* = .6), apathy (*r*_*s*_ = .12, *p* = .2), hallucinations (*r*_*s*_ = .11, *p* = .2), delusions (*r*_*s*_ = .09, *p* = .3) or altered personal boundaries (*r*_*s*_ = .12, *p* = .2). Five of the 15 patients showing altered phobic reactivity harboured a pathogenic mutation (three *C9orf72*, one *MAPT*, one *GRN*), and there was no evidence of an association between these mutations and changes in specific phobia in people with dementia (*r*_*s*_ = .12, *p* = .2).

Examining the directionality of change in those individuals with altered phobic reactivity, nine (including both healthy controls) had reduction or loss of a longstanding phobia while eight patients had developed a new phobia. Phobic alterations in patients had all developed since the onset of clinical illness. One patient with bvFTD exhibited a bidirectional alteration of phobic reactivity, with development of a new phobia (around water) and loss of premorbid longstanding claustrophobia. Of note, whereas there was no significant effect of diagnosis on loss of a previous phobia, only patients with FTD syndromes (eight cases, 8.8% of the combined FTD group) developed a new phobia; the probability of this did not differ between FTD syndromic groups (*p* = .9). The most common targets of altered phobic reactivity were spiders or insects (five cases) and heights (five cases); needles, snakes, water, choking and confined spaces were also represented. The two healthy controls had reduced phobic reactivity to spiders and heights, respectively. Examples of caregiver reports of patients' altered phobic responses are included in Table S3 in Supplementary Material online.

### 3.3 Neuroanatomical associations of altered phobic reactivity

**Fig. 1:**
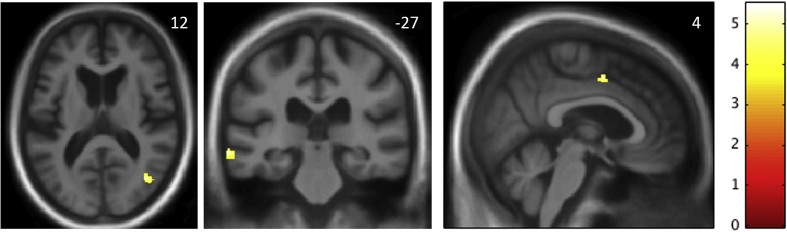
**Neuroanatomical associations of altered phobic reactivity.** The statistical parametric maps show areas of relative regional grey matter preservation associated with altered phobic reactivity in the behavioural variant frontotemporal dementia (bvFTD) syndromic group. Maps are based on the contrast between bvFTD subgroups with and without any change in phobic reactivity, thresholded for display purposes at *p* < .001 uncorrected for multiple voxel-wise comparisons over the whole brain; local maxima of clusters shown were all significant at *p* < .05 after family-wise error correction for multiple comparisons within pre-specified neuroanatomical regions of interest (see text and Figure S2). Maps are rendered on axial (left), coronal (middle) and sagittal (right) sections of the mean group template T1-weighted MR brain image; slice coordinates (mm) in Montreal Neurological Institute standard space are shown, and the right hemisphere is presented on the right in the axial and coronal sections. The colour bar codes voxel-wise T score values.

Maps of regional grey matter significantly associated with altered phobic reactivity within the bvFTD group are shown in Fig. 1. Altered phobic reactivity was associated with relative preservation of grey matter in left posterior middle temporal gyrus (cluster size 379 voxels, local maximum in Montreal Neurological Institute space [-68, −28, −11], t = 5.49, *p* = .02), right temporo-occipital junction (cluster size 77 voxels, local maximum [42, −70, 12], t = 3.97, *p* = .049) and right anterior cingulate gyrus (cluster size 24 voxels, local maximum [3, 3, 46], t = 4.72, *p* = .03), all thresholded at *p* < .05_FWE_ after correction for multiple voxel-wise comparisons within the pre-specified anatomical region of interest. There were no significant associations of altered phobic reactivity with regional grey matter loss at the prescribed threshold.

## 4 Discussion

We have shown that alterations in phobic reactivity are relatively common in major syndromes of FTD, particularly the behavioural variant, in comparison both to healthy older individuals and patients with AD. While alterations in phobic reactivity were bidirectional across the FTD cohort (and occasionally, in individual patients), development of a new phobia only occurred in patients with a syndrome of FTD. Altered phobic reactivity did not correlate with age, general disease factors or other neuropsychiatric symptoms, suggesting a degree of pathophysiological specificity. A neuroanatomical substrate for altered phobic reactivity in the bvFTD group was identified as relative preservation of grey matter in a distributed cingulo-temporo-occipital cortical network.

The increased prevalence of phobic changes in our FTD cohort is in line both with clinical experience and previous single-case studies (Clark et al., 2014). This finding foregrounds a symptom that has been largely overlooked by standard instruments in the field (Cummings et al., 1994; Goodarzi et al., 2019; Goyal, Bergh, Engedal, Kirkevold, & Kirkevold, 2017), while extending previous evidence that anxiety is a common symptom in dementia, especially FTD (Porter et al., 2003). More specifically, development of new phobic reactivity appears to be a hallmark of FTD, at odds both with the direction of phobic alterations observed in the present AD group and with previous work indicating that phobic reactivity tends to become attenuated in healthy older people (Byers et al., 2010; Chou et al., 2011; Grenier et al., 2019; Sigström et al., 2016; Stinson et al., 2007). Although we did not attempt to quantify the intensity of phobic reactivity in this study, it is noteworthy that (based on the reports of their caregivers; see Table S3) patients with FTD often exhibited marked phobic alterations with a disruptive impact in daily life: for example, a former arachnophobe now willingly handled spiders, while another patient developed new acrophobia of such severity that he even avoided watching tall buildings on television. This contrasts with the relatively subtle alterations in phobic awareness previously described in healthy older people (Grenier et al., 2011).

We interpret altered phobic reactivity in FTD as a signal of pathology involving the neural circuitry that appraises and assigns emotional value to salient (especially, aversive) sensory stimuli. This interpretation builds on two key lines of evidence for a more general abnormality of sensory object decoding and valuation in FTD syndromes, particularly bvFTD and svPPA. Patients with FTD have difficulty decoding ambiguous sensory signals (for example, those embodied in visual humour and abstract art (Clark et al., 2015; Cohen et al., 2016) and using context to resolve sensory incongruity (Clark et al., 2017). We would argue that phobic responses also entail processing of this kind, in the sense that snakes, spiders and high places (for example) are intrinsically salient, as they can indeed present a threat to well-being under certain circumstances, while a proportionate response to them demands contextual processing (i.e., that particular object is innocuous, physically remote or otherwise constrained from causing the subject actual harm). In addition, while reduced behavioural and physiological sensitivity to aversive stimuli is increasingly recognised in FTD syndromes (Hoefer et al., 2008; Perry et al., 2017), FTD is also known to be associated with ‘bivalent’ alterations to a diverse plethora of biologically salient sensory stimuli, including variably heightened and/or attenuated responses to food, pain, sounds, ambient temperature, sex and inter-personal emotional signals (Ahmed et al., 2015; Clark & Warren, 2016; Fletcher et al., 2015a, Fletcher et al., 2015b; Thompson et al., 2016). The bidirectional phobic alterations described across the present FTD cohort illustrate this broader theme, and suggest a fundamental deficit in matching sensory templates to behavioural outputs, perhaps due to an inability to regulate the ‘gain’ of sensory salience and/or reward coding (Clark & Warren, 2016; Perry et al., 2017).

This interpretation is supported by the present neuroanatomical evidence, implicating brain regions previously shown to mediate contextual decoding, salience processing and reward valuation in FTD as well as the healthy brain (Clark et al., 2015; Cohen et al., 2016; Perry & Kramer, 2015; Seeley et al., 2009). More specifically, the relative preservation of grey matter in anterior cingulate and higher order visual association cortices in our FTD patients who exhibited phobic alterations accords with previous structural and functional imaging studies of phobic responses (Caseras et al., 2010; Del Casale et al., 2012; Hermann et al., 2009; Hilbert, Evens, Isabel Maslowski, Wittchen, & Lueken, 2015; Ipser et al., 2013; Lange et al., 2019; Linares et al., 2012; Mobbs et al., 2010; Rauch et al., 2004; Stefanescu et al., 2018). However, in contrast to the situation in the otherwise healthy brain, FTD constitutes a ‘lesion model’ for the development of altered phobic reactivity: the present findings suggest that cingulo-temporo-occipital circuitry may play a critical role in modulating or regulating phobic reactivity, opening a novel window on the neural mechanisms that mediate phobias and strong fear responses more generally. The absence of an amygdalar correlate here may reflect the lack of a parametric phobic threat measure in our symptom survey (Mobbs et al., 2010).

This study raises several caveats that should motivate further work. The findings should be extended in larger patient cohorts, ideally with pathological correlation; it is likely the present study was under-powered to detect differences in the profiles of phobic reactivity that may have further stratified FTD syndromes and/or genetic subgroups. It would also be of interest to assess the longitudinal evolution of altered phobic reactivity. In tandem with this, there is a need to develop standardised instruments to detect and quantify phobic responses and to assess their daily life impact in cognitively impaired populations. A related issue concerns the reporting of phobic reactions: here, information was obtained about healthy controls' own reactions but about patients' reactions via their caregivers. Ideally, a uniform reporting protocol would be used both in patients and healthy controls and it would be of interest to compare patients' own awareness of phobic reactivity with their caregivers' reports. The phobic targets here were generally banal and similar to those commonly provoking specific phobia in the healthy population: although the small case numbers here precluded such an analysis, it would be of interest to determine whether particular molecular pathologies might show differential phobic phenomenology. For example, one might predict a predilection for phobic alterations linked to personal boundaries in association with *C90rf72* mutations (Downey et al., 2014). The pathophysiological mechanisms that mediate phobic alterations will only be fully delineated by functional neuroimaging techniques that can examine large-scale brain network connectivity changes and by correlation with autonomic responses. The latter will be particularly pertinent in FTD, in which abnormal physiological processing of sensory signals (Marshall, Hardy, Allen, et al., 2018; Marshall, Hardy, Russell, et al., 2018; Marshall et al., 2017) and abnormal fear conditioning (Hoefer et al., 2008) have emerged as significant issues that could clearly affect the subjective experience of fear in these patients. Besides abnormal salience coding, there are potentially several other, non-exclusive candidate mechansisms that could lead to altered phobic responses (including, for example, impaired understanding of phobic objects, and loss of insight into the nature and appropriateness of one's own fear response). Indeed, the neural mechanisms that mediate attenuated versus heightened phobic reactivity might, at least in prnciple, themselves be separable. These mechanisms are not resolved in this study and are likely to require connectivity-based techniques to tease apart, if ‘bidirectional’ behavioural changes in FTD arise from shared neural circuitry (Clark & Warren, 2016). Furthermore, while we did not find evidence for a straightforward linkage here, is not yet clear how the cognitive and neural processes that promote phobic alterations might interact with the processes subserving psychosis and related neuropsychiatric phenomena in patients with FTD and other dementias (Cipriani, Danti, Vedovello, Nuti, & Lucetti, 2014; Downey et al., 2014). Finally, more information is required concerning phobic changes in the healthy elderly, in order to interpret disease-associated phobic phenomena correctly.

Taking these caveats into account, our findings have identified a novel behavioural phenomenon in FTD with both clinical and neurobiological implications. Clinically, prominent changes in phobic reactivity may corroborate the clinical diagnosis of FTD; moreover, the appearance of new phobias can disrupt daily routines and cause distress in its own right, while loss of phobic reactivity could potentially confer increased vulnerability to harm, if in fact this signals a more general attenuation of fear responses. Neurobiologically, altered phobic reactivity constitutes a novel paradigm for investigating the brain mechanisms that support the decoding of salient (in particular, aversive) sensory stimuli in neurodegenerative disease and the experience of strong fear more broadly.

## Author contributions

**Daniel Jimenez:** Conceptualisation, Methodology, Investigation, Formal analysis, Writing- Original draft preparation, Writing- Reviewing and Editing.

**Rebecca Bond:** Methodology, Investigation, Writing- Reviewing and Editing.

**Mai-Carmen Requena-Komuro:** Methodology, Investigation, Writing- Reviewing and Editing.

**Harri Sivasathiaseelan:** Methodology, Investigation, Writing- Reviewing and Editing.

**Charles Marshall:** Methodology, Investigation, Writing- Reviewing and Editing.

**Lucy Russell:** Investigation, Writing- Reviewing and Editing.

**Caroline Greaves:** Investigation, Writing- Reviewing and Editing.

**Katrina Moore:** Investigation, Writing- Reviewing and Editing.

**Ione Woollacott:** Investigation, Writing- Reviewing and Editing.

**Rachelle Shafei:** Investigation, Writing- Reviewing and Editing.

**Chris Hardy:** Methodology, Investigation, Formal analysis, Writing- Reviewing and Editing.

**Jonathan Rohrer:** Funding acquisition, Conceptualisation, Writing- Reviewing and Editing, Supervision.

**Jason Warren:** Funding acquisition, Conceptualisation, Methodology, Writing- Reviewing and Editing, Supervision.

## Open practices

The study in this article earned an Open Materials badge for transparent practices.

## Declaration of Competing Interest

None.
