# Supplementary material for: Altered phobic reactions in frontotemporal dementia: A behavioural and neuroanatomical analysis
Source: Cortex. 2020 Sep;130:100–10. doi: 10.1016/j.cortex.2020.05.016 (PMC7447974; doi:10.1016/j.cortex.2020.05.016)
Supplement: Multimedia component 1 [file mmc1.doc]

**SUPPLEMENTARY MATERIAL: Altered phobic reactions in frontotemporal dementia: a behavioural and neuroanatomical analysis, by DA Jimenez et al.**

**Table S1.** Survey used to record general neuropsychiatric and specific phobia symptoms

| **Domain** | **Question** | **Examples** | **Information recorded** |
| --- | --- | --- | --- |
| **General neuropsychiatric** | Does s/he currently show any of the following symptoms: |  |  |
|  | Apathy | *Lack of drive, motivation or initiative, particularly for activities they previously enjoyed or did regularly* | Yes / No |
|  | Hallucinations | *Seeing or hearing people, animals or other things that are not really there* | Yes / No  If yes, details |
|  | Delusions | *Odd or incorrect, strongly held beliefs that cannot be altered by contrary evidence or reasonable arguments* | Yes / No  If yes, details |
|  | Anxiety | *Persisting sense of unease, dread or apprehension without a specific cause* | Yes / No |
|  | Agitation | *Restless, upset or aggressive behaviour without a specific cause* | Yes / No |
|  | Altered self boundaries | *Dislike of being approached or touched by others; standing ‘too close’ or unwelcome touching of others* | Yes / No |
| **Phobic reactivity** | If s/he has ever reported or shown evidence of a phobia | *An intense, fear of a specific object or situation, out of proportion to any actual danger and which had led them to try to avoid the object or situation* |  |
|  | What was the phobic object or situation? | *Please list all / any* | Details |
|  | Has there been any change in the type or strength of the phobia during the past 10 years? |  | Yes / No |
|  | If so, has the phobia become more or less evident? |  | Increased / Decreased |
|  | Is there a new phobia? |  | Details |
|  | Do you have any other comments about the phobia? |  | Details |

The survey was completed by each patient’s primary caregiver or by healthy controls themselves. We assessed the presence or absence of neuropsychiatric symptoms anticipated to be potentially relevant to the development and/or expression of altered phobic reactivity. Cases with phobic symptoms that could reflect social anxiety (‘social phobia’) or a generalised anxiety disorder were excluded. See text for further details.

**Table S2.** Participant characteristics by diagnosis and presence vs absence of altered phobic reactivity

| **Characteristic** | **Controls** | | **bvFTD** | | **svPPA** | | **nfvPPA** | | **AD** | |
| --- | --- | --- | --- | --- | --- | --- | --- | --- | --- | --- |
|  | No | Yes | No | Yes | No | Yes | No | Yes | No | Yes |
| **General** |  |  |  |  |  |  |  |  |  |  |
| Total, n (%) | 53 (96.4) | 2 (3.6) | 38 (82.6) | 8 (17.9) | 17 (85) | 3 (15) | 22 (88) | 3 (12) | 28 (96.6) | 1 (3.5) |
| Gender (F:M) | 24:29 | 1:1 | 12:26 | 1:7 | 7:10 | 2:1 | 11:11 | 1:2 | 14:14 | 1:0 |
| Age at assessment, years | 64.4 (7.0) | 77.1 | 64.4 (6.6) | 65.4 (5.0) | 66.0 (7.0) | 64.8 (9.1) | 69.1 (8.0) | 70.8 (5.9) | 71.2 (7.8) | 63.4 |
| Symptom duration, years | NA | NA | 6.1 (3.7) | 9.0 (8.0) | 5.3 (2.4) | 6.1 (1.9) | 5.0 (4.7) | 3.2 (1.0) | 6.8 (3.5) | 4.4 |
| MMSE ( /30) | 29.4 (0.9) | 29.5 (0.7) | 23.9 (5.7) | 25.8 (5.0) | 24.5 (4.6) | 17 (14.1) | 21.0 (8.4) | 19.7 (2.1) | 18.9 (5.9) | 28 |
| **Neuropsychiatric symptoms** |  |  |  |  |  |  |  |  |  |  |
| Apathy, n (%)^a^ | 2 (3.8) | 0 | 30 (79.0) | 7 (87.5) | 8 (47.1) | 1 (33.3) | 13 (59.1) | 1 (33.3) | 19 (67.9) | 1 (100) |
| Hallucinations,  n (%)^a^ | 0 | 0 | 9 (23.7) | 2 (25) | 0 | 1 (33.3) | 0 | 0 | 4 (14.3) | 0 |
| Delusions, n %)^a^ | 0 | 0 | 14 (36.8) | 3 (37.5) | 4 (23.5) | 0 | 2 (9.1) | 1 (33.3) | 4 (14.3) | 0 |
| Anxiety, n (%)^a^ | 3 (5.7) | 0 | 15 (39.5) | 4 (50) | 10 (58.8) | 1 (33.3) | 16 (72.7) | 1 (33.3) | 15 (53.6) | 1 (100) |
| Agitation, n (%)^a^ | 0 | 0 | 13 (34.2) | 3 (37.5) | 4 (23.5) | 0 | 3 (13.6) | 0 | 2 (7.1) | 0 |
| Altered personal boundaries, n (%)^b^ | 0 | 0 | 8 (21.1) | 1 (12.5) | 5 (29.4) | 0 | 2 (9.1) | 1 (33.3) | 2 (7.1) | 1 (100) |

Mean (standard deviation) values are shown unless otherwise indicated. Key: AD, patient group with typical Alzheimer’s disease; bvFTD, patient group with behavioural variant frontotemporal dementia; Controls, healthy control group; MMSE, Mini-Mental State Examination score; NA, not applicable; nfvPPA, patient group with non-fluent variant PPA; No, no phobic alteration reported; svPPA, patient group with semantic variant primary progressive aphasia; Yes, phobic alteration reported. ^a^21 missing values (14 controls, four bvFTD, one svPPA, two nfvPPA); ^b^ 42 missing values (14 bvFTD, 22 bvFTD, one svPPA, five nfvPPA).

**Table S3.** Selected caregiver reports of changes in patients’ phobic reactions

| **Age/gender** | **Diagnosis** | **Direction of change** | **Phobic object or situation** | **Caregiver comments** |
| --- | --- | --- | --- | --- |
| 61/M | bvFTD | Loss | Heights | He recently enjoyed several vertiginous rollercoasters at theme park which he certainly would not have done previously |
| 61/M | bvFTD | Loss/new | Flying, heights,  confined spaces (loss); water (new) | His previous fear of flying has gone. He is no longer worried by heights and confined spaces. He was always a strong swimmer but now he sits on the edge of the swimming pool willing himself to get in the water. |
| 73/M | bvFTD | New | Snakes | This was always my [his wife’s] phobia and he never understood it. |
| 65/M | bvFTD | New | Needles | At his most recent hospital appointment he had to be physically restrained when a blood sample was required. |
| 67/F | svPPA | Loss | Spiders | Recently she has picked up spiders in the house, with her bare hands. |
| 72/F | svPPA | New | Needles | She started shivering when asked to take her coat off in clinic – she is now phobic of needles. |
| 75/M | nfvPPA | Loss | Confined spaces | He will now have an MRI scan which he would not have previously |
| 64/F | nfvPPA | New | Heights | She is now scared of slopes when skiing, panicked mounting a horse to go riding |
| 73/M | nfvPPA | New | Heights | He has developed a paralysing fear of tall buildings over the past few years - he refuses to enter or approach high elevations and becomes anxious even seeing a tall building on TV. |
| 63/F | AD | Loss | Flying insects | She has always had a fear of these insects, but this has now disappeared. |

Key: AD, typical Alzheimer´s disease; bvFTD, behavioural variant frontotemporal dementia; nfvPPA, non-fluent primary progressive aphasia; svPPA, semantic variant primary progressive aphasia.

**Figure S1.** **Pre-specified regions of interest for VBM.** Representative coronal (left), sagittal (middle) and axial (right) sections are shown for the neuroanatomical volumes selected for multiple voxel-wise comparison correction in voxel-based morphometric, region-of-interest analyses based on prior anatomical hypotheses. **A,** bilateral amygdala; **B,** bilateral cingulate gyrus, anterior division; **C,** bilateral insular cortex; **D,** composite region covering temporo-occipital junction. These regions were customised from the Oxford/Harvard brain maps to fit the group mean template brain image.
